# Comparison of Sociodemographic and Radiographic Features in Distal Radio Fracture Treatment: Hand Surgeons versus Non-specialists *

**DOI:** 10.1055/s-0043-1776017

**Published:** 2024-03-21

**Authors:** Rafael Bulyk Veiga, Renê Hobi, Ricardo Pereira Marot, Gustavo Zeni Schuroff, Roberto Luiz Sobania, Ivan Killing Kuhn, Ana Lucia Campos Faccioni

**Affiliations:** 1 Departamento de Ortopedia e Traumatologia, Hospital de Clínicas, Universidade Federal do Paraná, Curitiba, PR, Brasil; 2 Departamento de Ortopedia e Traumatologia, Hospital do Trabalhador, Universidade Federal do Paraná, Curitiba, PR, Brasil

**Keywords:** radius fractures, wrist injuries, fracture reduction, treatment result, evaluation of results of therapeutic interventions

## Abstract

**Objective:** This study evaluated sociodemographic and radiographic features of patients with distal radial fractures treated at a trauma hospital in southern Brazil, comparing those treated by hand surgery specialists (group 1) and non-specialists (group 2).

**Methods:** This study consists of a retrospective cohort of 200 patients treated in 2020. After reviewing medical records and radiographs, the following parameters were analyzed: age, gender, trauma mechanism, laterality, associated comorbidities and fractures, fracture classification (AO), radial height, radial inclination, and volar inclination. Comparison of the two groups used the Student t-test, chi-square test, or Fisher exact test.

**Results:** Most subjects were women (54%), sustained low-energy traumas (58%), and were left-handed (53%). Group 1 had a lower mean age (50.2 years); most of their subjects sustained high-energy trauma (54%) and had type C fractures (73%); type A fractures prevailed in group 2 (72%). Radiographs showed a significant difference regarding the mean radial inclination (21.5° in group 1 and 16.5° in group 2 [ *p* < 0.001] in women, and 21.3° in group 1 and 17° in group 2 [ *p* < 0.001] in men) and volar inclination (10.1° and 12.8° in groups 1 and 2, respectively [ *p* < 0.001]). In addition, the absolute number of cases with reestablished anatomical parameters per the three evaluated variables was also significantly different; all parameters were better in group 1.

**Conclusion:** Hand surgeons treated the most severe fractures and had the best radiographic outcomes.

## Introduction

Distal radial fracture (DRF) is the most common upper limb fracture, 1 2 3 4 5 6 7 8 9 10 representing 10 to 25% of injuries in orthopedic emergencies. 11 12 13 14 15 It has a bimodal distribution and is associated with high-energy trauma in young people and low-energy trauma in elderly patients. 2 4 7 The incidence of DRF is growing due to increased life expectancy and osteoporosis. 4 6 7 9

Distal radial fracture is the second most common fracture in elderly subjects, 16 compromising their functionality and causing disability. 13 It also impacts younger people, 12 influencing them financially and professionally, becoming a public health problem. 16

The goal of the orthopedist is to restore wrist function and mobility. 2 3 16 However, there is no consensus on the ideal treatment, considering age, activity level, occupation, bone quality, fracture type, and quality of life. 1 2 10 12 16

Assessing treatment outcomes is difficult because therapeutic success is multifactorial. The orthopedist can interfere with the restoration of radiographic parameters. 5 7 Therefore, their restoration is essential to achieve satisfactory functional outcomes. 4 11 13 17

The most significant radiographic parameters in DRF are radial height (RH), radial inclination (RI), and volar inclination (VI). 4 5 11 However, there is no consensus on the ideal values of those. 10 We considered the following values: RH, 11.6 mm ± 1.6 mm; RI, 24.7° ± 2.5° in women and 22.5° ± 2.1° in men; and VI, 11.2° ± 4.6°. 18

Conservative treatment has poor outcomes, especially in young people with joint fractures. 1 2 In elderly patients, it does not lead to reduction; however, there is no difference in functional capacity after conservative or surgical treatment. 4 11 12

The alternatives for surgical treatment include Kirschner wires (KW), external fixators (EF), and open reduction with internal fixation (ORIF). 2 4 Kirschner wires and EF are less invasive, less complex, and have a lower financial impact. However, ORIF is the most effective treatment for unstable and joint fractures 2 as it promotes stable fixation and early mobilization, 4 15 better functional and satisfaction outcomes, 6 8 9 16 17 and lower osteoarthritis rates. 15

Loss of RH is a relevant factor affecting functional capacity, which may lead to pain, osteoarthritis, instability, and limited pronosupination. 17 Moreover, VI with a dorsal deviation above 20° increases the osteoarthritis risk. 4 16 Patient satisfaction depends on posttreatment pain intensity, wrist function, and mobility. 19 20

It is uncertain whether orthopedists receive sufficient training to treat this type of fracture. A recent study with heads of hand surgery programs demonstrated that practicing ORIF techniques is essential in training but that the exposure gap is up to 53%. 21

Given the deficit in training and the topic's significance, this study aimed to evaluate sociodemographic and radiographic variables in DRF and compare the outcomes obtained by hand surgery specialists and non-specialists

## Materials and Methods

This is a retrospective cohort study of patients with DRF treated at a trauma hospital in southern Brazil.

The sample consisted of 200 patients treated from January 1, 2020, to December 31, 2020. Hand surgery specialists treated half of these cases (group 1), while non-specialist orthopedists treated the other half (group 2).

The study included the last 100 patients treated by each group in the mentioned period. Other inclusion criteria were age over 18 and unilateral fractures treated within 1 week of the trauma.

The exclusion criterion was to not meet the inclusion criteria. No patient was excluded due to lack of data in the medical record.

The variables analyzed in the medical records included age, gender, trauma mechanism, laterality, associated comorbidities and fractures, and treatment method.

Radiographs from the day of trauma were the basis for fracture classification using the Arbeitsgemeinschaft für Osteosynthesefragen (AO) system. 22

One week after immobilization with plaster casts or the surgical procedure, we requested follow-up radiographs to calculate RH, RI, and VI.

The criteria for surgical treatment indication included signs of fracture instability (dorsal deviation >20°, comminution of the dorsal cortex, joint involvement, associated ulnar fracture, and radius shortening > 9 mm). 1

The same researcher collected the radiographic parameters. Radial inclination was the angle between a line perpendicular to the line of the long axis of the radial diaphysis and a line connecting the radial styloid to the ulnar radial corner in an anteroposterior radiograph. Radial height was the difference in axial length between the tip of the radial styloid and the ulnar radial corner in an anteroposterior radiograph. Volar inclination was the angle between a line perpendicular to the longitudinal axis of the radius and a line formed by the connection of the volar apex and the dorsal edges of the radius in a lateral radiograph. 18

Normal parameters were the following: RH, 11.6 mm ± 1.6 mm; RI, 24.7° ± 2.5° in women, and 22.5° ± 2.1° in men; and VI, 11.2° ± 4.6°. 18

A microcomputer processed the data using a Microsoft Excel 2016 (Microsoft Corp., Redmond, WA, USA) database. Mean, minimum, maximum, and standard deviation (SD) values described quantitative variables. Frequencies and percentages summarized qualitative variables. A Student t-test compared quantitative variables between groups. The chi-square or Fisher exact test evaluated the association between two qualitative variables. Statistical analysis was performed using the SPSS Statistics for Windows, version 18.0 (SPSS Inc., Chicago, IL, USA), and the significance level was set at 5%.

The Research Ethics Committee approved this study under opinion No. 5,310,541.

## Results

The global sample ( Table 1 ) consisted mostly of women (54%) and presented a mean age of 50.7 years. The prevalence of DRF was higher on the left side (53%). Low-energy traumas were the most common. Among them, 86 patients had type A (extra-articular), 25 type B (partial articular), and 89 type C (complete articular) fractures. The most used treatment methods were KW (62.5%), followed by ORIF, conservative treatment, and EF.

**Table 1:** Data from patients treated by hand surgery specialists and non-specialists

		Specialist (group 1)	Non-specialist (group 2)	*p*
Gender	**Male (n)**	48	44	0.57
	**Female (n)**	52	56	
Age	**Mean (years)**	50.2	51.3	0.64
Mechanism	**Low energy (n)**	46	70	0.001
	**High energy (n)**	54	30	
Side	**Right (n)**	49	45	0.571
	**Left (n)**	51	55	
Comorbidity	**Yes (n)**	28	33	0.443
	**No (n)**	72	67	
Associated fracture	**Yes (n)**	24	12	0.027
	**No (n)**	76	88	
AO classification	**A (n)**	14	72	< 0.001
	**B (n)**	13	12	
	**C (n)**	73	16	
Radial height	**Mean (mm)**	11.04	10.8	0.299
	**Loss (n)**	11	35	< 0.001
	**Gain (n)**	3	12	
Radial inclination	**Women (°)**	21.5	16.5	< 0.001
	**Men (°)**	21.3	17	< 0.001
	**Loss (n)**	51	87	< 0.001
	**Gain (n)**	3	2	
Volar inclination	**Mean (°)**	10.1	12.8	< 0.001
	**Loss (n)**	16	1	< 0.001
	**Gain (n)**	5	15	
Treatment	**Kirschner wire (n)**	47	78	–
	**Plate and screw (n)**	39	15	
	**Cast (n)**	9	5	
	**External fixation (n)**	5	2	

Abbreviations: n, Number of cases; mm, millimeter; ° - degrees, *p* – group comparison.

For radiographic parameters, mean RH was 10.92 mm, mean RI was 18.9° in women and 19.5° in men, and mean VI was 11.4°. Of these patients, 23% lost and 7.5% gained RH; 69% lost and 2.5% gained RI; 8.5% lost and 10% gained VI.

Most patients treated by hand surgery specialists (group 1 - Table 1 ) presented high-energy traumas (54%) and type-C fractures (73%). Their radiographic parameters were RH, 11.04 mm (range, 8–15.36; standard deviation [SD], 1.17), RI, 21.5° (range, 16–27; SD, 2.4) in women and 21.3° (range, 17–28; SD, 2.7) in men, and VI, 10.1° (range, 2–22; SD, 3.8). Of these, only 11 cases presented RH loss and 3 had RH gain; 51 cases had RI loss and 3, RI gain; and 16 presented VI loss and 5, VI gain.

Most subjects treated by orthopedists who are not specialists in hand surgery (group 2 - Table 1 ) sustained a low-energy trauma (70%) and presented type-A fractures (72%). In this group, mean RH was 10.8 mm (range, 5.95–15.71; SD, 1), RI was 16.5° (range, 10–29; SD, 3.8) in women and 17° (range, 12–25; SD, 2.97) in men, and VI was 12.8° (range, 3–27; SD, 3.5). Of these patients, 35 lost and 12 gained RH; 87 lost and 2 gained RI, and 1 lost and 15 gained VI.

Group comparison ( Table 1 ) revealed more high-energy traumas (54 versus 30 cases, *p* <0.001) and associated fractures (24 × 12 cases, *p* = 0.027) in group 1. Group 2 presented more type-A fractures (72%), while group 1 had more type-C fractures (73%).

A comparison of radiographic parameters showed no statistical difference for mean RH (11.04 versus 10.8, *p* = 0.29), but mean RI and VI were better in group 1 (mean RI of 21.5° versus 16.5° [ *p* < 0.001] in women and 21.3° versus 17° [ *p* <0.001] in men; mean VI of 10.1° and 12.8° in group 1 and 2, respectively [ *p* <0.001]). When we evaluated the absolute number of cases that did not reach the radiographic parameters, there was a statistical difference between them, with the results of group 2 being worse ( *p* < 001).

As for fractures ( Table 2 ), type A had better RI and VI results; mean RI was 22.2° versus 16.5° ( *p* < 0.001) in women and 22° versus 17.7° ( *p* = 0.011) in men, and mean VI was 10.4° versus 12.5° ( *p* = 0.017).

**Table 2:** Comparison of type-A fractures between groups treated by hand surgery specialists and non-specialists

		Type A		
		Specialist (group 1)	Non-specialist (group 2)	*p*
Gender	**Male (n)**	4	31	0.313
	**Female (n)**	10	41	
Age	**Mean (years)**	49.1	51.8	0.61
Mechanism	**Low energy (n)**	8	52	0.261
	**High energy (n)**	6	20	
Side	**Right (n)**	8	31	0.333
	**Left (n)**	6	41	
Comorbidity	**Yes (n)**	3	22	0.491
	**No (n)**	11	50	
Associated fracture	**Yes (n)**	2	8	0.735
	**No (n)**	12	64	
Radial height	**Mean (mm)**	10.91	10.73	0.654
	**Loss (n)**	1	26	–
	**Gain (n)**	0	9	
Radial inclination	**Women (°)**	22.2	16.5	< 0.001
	**Men (°)**	22	17.7	0.011
	**Loss (n)**	8	63	–
	**Gain (n)**	0	2	
Volar inclination	**Mean (°)**	10.4	12.5	0.017
	**Loss (n)**	1	0	–
	**Gain (n)**	0	10	
Treatment	**Kirschner wire (n)**	12	65	–
	**Plate and screw (n)**	1	2	
	**Cast (n)**	0	5	
	**External fixation (n)**	1	0	

Abbreviations: n, Number of cases; mm, millimeter; ° - degrees, *p* – group comparison.

Type-B fractures ( Table 3 ) showed better results only for RI in women from group 1 (21° versus 15.8°, *p* = 0.015). Type-C fractures ( Table 4 ) had better results for RI (21.3° versus 17.3° [ *p* = 0.001] in women and 21.4° versus 16.7° [ *p* < 0.001] in men) and VI (10° versus 13.1° [ *p* = 0.02]) in group 1; ORIF was the preferred treatment for these fractures in both groups.

**Table 3:** Comparison of type B fractures between groups treated by hand surgery specialists and non-specialists

		Type B		
		Specialist (group 1)	Non-specialist (group 2)	*p*
Gender	**Male (n)**	9	4	0.073
	**Female (n)**	4	8	
Age	**Mean (years)**	41.8	48.3	0.422
Mechanism	**Low energy (n)**	3	8	0.028
	**High energy (n)**	10	4	
Side	**Right (n)**	7	6	0.848
	**Left (n)**	6	6	
Comorbidity	**Yes (n)**	3	5	0.319
	**No (n)**	10	7	
Associated fracture	**Yes (n)**	5	1	0.078
	**No (n)**	8	11	
Radial height	**Mean (mm)**	11.32	11.19	0.85
	**Loss (n)**	0	3	–
	**Gain (n)**	1	2	
Radial inclination	**Women (°)**	21	15.8	0.015
	**Men (°)**	20.4	18.3	0.235
	**Loss (n)**	6	10	–
	**Gain (n)**	1	0	
Volar inclination	**Mean (°)**	10.7	12.3	0.145
	**Loss (n)**	1	0	–
	**Gain (n)**	1	1	
Treatment	**Kirschner wire (n)**	3	8	–
	**Plate and screw (n)**	4	3	
	**Cast (n)**	6	0	
	**External fixation (n)**	0	1	

Abbreviations: n, Number of cases; mm, millimeter; ° - degrees, p – group comparison.

**Table 4:** Comparison of type-C fractures between groups treated by hand surgery specialists and non-specialists

		Type C		
		Specialist (group 1)	Non-specialist (group 2)	*p*
Gender	**Male (n)**	35	9	0.547
	**Female (n)**	38	7	
Age	**Mean (years)**	51.9	51.6	0.947
Mechanism	**Low energy (n)**	35	10	0.292
	**High energy (n)**	38	6	
Side	**Right (n)**	34	8	0.804
	**Left (n)**	39	8	
Comorbidity	**Yes (n)**	22	6	0.566
	**No (n)**	51	10	
Associated fracture	**Yes (n)**	17	3	0.694
	**No (n)**	56	13	
Radial height	**Mean (mm)**	11.02	10.8	0.649
	**Loss (n)**	10	6	–
	**Gain (n)**	2	1	
Radial inclination	**Women (°)**	21.3	17.3	0.001
	**Men (°)**	21.4	16.7	< 0.001
	**Loss (n)**	37	14	–
	**Gain (n)**	2	0	
Volar inclination	**Mean (°)**	10	13.8	0.02
	**Loss (n)**	14	1	–
	**Gain (n)**	4	4	
Treatment	**Kirschner wire (n)**	32	5	–
	**Plate and screw (n)**	34	10	
	**Cast (n)**	3	0	
	**External fixation (n)**	4	1	

Abbreviations: n, Number of cases; mm, millimeter; ° - degrees, *p* – group comparison.

## Discussion

The literature shows that DRF mainly affects women, 3 4 5 6 7 8 9 10 11 12 13 14 15 17 which is consistent with this study. The average age was 50.7 years, lower than that in most studies, 2 3 4 5 6 7 9 12 13 15 18 and few reported average values lower than that. 8 10

Distal radial fracture has a bimodal distribution, with high-energy traumas associated with younger people (< 60 years). 4 8 This contrasts with our findings since the mean age of our patients was lower than 60, but most cases were due to low-energy trauma. This divergence may result from the wide age variation in our sample, ranging from 19 to 100 years old.

Laterality is a significant factor because of the impact on the subject's daily activities. 12 13 Some authors demonstrated a predominance in the non-dominant limb, 3 8 13 while others reported the dominant limb as most affected. 5 6 11 14 Some authors also define laterality in terms of right and left. In these studies, fractures occurred predominantly in the left limb, 2 3 9 15 which is also consistent with our findings.

In our study, only 30.5% of the patients had comorbidities, corroborating other papers noting a low comorbidity rate. 5 10 Eighteen percent of our patients presented associated fractures; the literature reports some kind of associated injury in 39 to 84% of cases, but it does not specify the percentage of concomitant fractures. 12

Regarding the type of fracture, 86 cases were type-A fractures, 25 were type B, and 89 were type-C fractures. This finding is consistent with other studies, which showed a higher rate of type-A 11 12 and type C-fractures. 4 6 9 15

The literature shows that the most used treatments are ORIF and conservative method. Those who prefer conservative treatment 10 11 12 argue that this is the main therapeutic method considering the greater bone remodeling in young people and the low functional demand in elderly subjects. 10 Authors preferring ORIF 2 3 6 17 state that it provides better fracture reduction, allowing early mobility and better functional outcomes. 6 8 9 14 15 16 17 In addition, hand surgeons are more likely to use ORIF. 16 Despite this, in our study, KW was the preferred treatment (62.5%), possibly due to the high number of extra-articular fractures and elderly patients, for whom a less invasive method is ideal. Furthermore, most studies occurred in developed countries, where fixation with locked plates replaced less aggressive methods; 10 therefore, we may still be in a transitional period.

Most cases from group 1 resulted from high-energy trauma. The mean age in this group was slightly lower, consistent with the idea that high-energy traumas are more frequent in younger people. Furthermore, group 1 presented a predominance of type-C fractures (73%). In contrast, group 2 had a higher number of type-A fractures (72%), probably because hand surgery specialists treated more complex cases. 4

As for radiographic parameters, the literature differs in outcome presentations; some papers report absolute values after treatment, 2 3 5 8 14 17 while others show the pre and posttreatment variation. 4 9 11 12 We observed a mean RH of 10.92 mm, which is consistent with the literature 3 5 8 14 17 In our sample, RI was 18.9° in women and 19.5° in men, and VI was 11.4°, lower than the reported values. 2 3 4 5 7 8 9 11 14 The fixation method may account for this difference, even though studies have shown no differences in radiographic outcomes associated with the fixation method. 1.2

Furthermore, there is a contradiction in the relationship between radiographic outcomes and function, especially in elderly people with lower demands. 3 4 5 6 7 11 13 However, several factors influence the therapeutic outcome, including fracture reduction, which the orthopedist can interfere with. 5 7 Therefore, reduction is essential to achieve better functional outcomes, 4 11 13 17 a lower osteoarthritis rate, 6 15 and better mobility. 4

A study demonstrated that posttraumatic osteoarthritis relates to radiographic alterations in RI and VI. 15 Another study showed that only 54% of the cases present restoration of all radiographic parameters; RH loss accounts for the worst functional outcomes, loss of mobility, decreased grip strength, and chronic pain. 4 6 17

A comparison of radiographic parameters between the groups revealed that group 1 had more cases with restored normal parameters and better RI and VI mean values. As far as we know, the literature has no study with a comparison similar to ours. One paper reported no difference in outcomes when considering the surgeon's experience. 4 Keeping in mind that the best reduction can lead to the best functional and satisfaction outcomes, we can suggest that patients treated by hand surgery specialists presented better outcomes in our study.

As for fracture type, type-A fractures had a higher percentage of patients with recovered RH in group 1; in addition, mean IR and VI values were better in this group. Type-B fractures had better outcomes in group 1 for mean RI in women. Type-C fractures showed better results in IR and VI when treated by hand surgery specialists.

## Study Limitations

In addition to being a retrospective study, other limitations included the lack of consensus on the normal radiological parameters 9 and the fact that radiographs were taken 1 week after treatment, not considering potential complications or loss of long-term reduction. Most cases treated by hand surgery specialists are more complex, hindering the collection of homogeneous samples between groups. Although low-demand patients accepted a higher deviation of the distal radius fracture for nonsurgical treatment, we did not evaluate the pre and posttreatment functional degrees. Therefore, further functional and satisfaction studies are required to determine treatment outcomes since fracture reduction is only one of the pillars for therapeutic success.

## Conclusion

This study demonstrated that hand surgery specialists treat the most complex DRF cases. Even in more severe cases, specialists presented better radiographic outcomes when compared to non-specialist orthopedists.

Since these outcomes are directly linked to better functional outcomes and patient satisfaction, orthopedists must prepare themselves to achieve the best radiographic results.
