# Supplementary material for: Comparison of Sociodemographic and Radiographic Features in Distal Radio Fracture Treatment: Hand Surgeons versus Non-specialists
Source: Rev Bras Ortop (Sao Paulo). 2024 Mar 21;59(1):e46–53. doi: 10.1055/s-0043-1776017 (PMC10957272; doi:10.1055/s-0043-1776017)
Supplement: Related texts

# Research article: Comparação de características sociodemográficas e radiográficas no tratamento de fratura de rádio distal: Cirurgiões de mão versus não especialistas *

**Keywords:** fraturas do rádio, traumatismos do punho, redução de fratura, resultado do tratamento, avaliação de resultados de intervenções terapêuticas

## Abstract

**Objetivo:** Avaliar as características sociodemográficas e radiográficas dos pacientes com fratura de rádio distal tratados em um hospital de trauma no sul do Brasil, comparando os casos tratados pelos especialistas em cirurgia da mão (grupo 1) com aqueles tratados por não especialistas (grupo 2).

**Métodos:** Coorte retrospectiva realizada com 200 pacientes, no ano de 2020. Por meio da revisão de prontuários e radiografias, analisaram-se: idade, sexo, mecanismo de trauma, lateralidade, presença de comorbidades e fraturas associadas, classificação da fratura (AO), altura radial, inclinação radial e, inclinação volar. Comparou-se os dois grupos por meio do teste t de Student, qui-quadrado ou exato de Fisher.

**Resultados:** A maioria era de pacientes do sexo feminino (54%), traumas de baixa energia (58%) e lateralidade esquerda (53%). O grupo 1 apresentou média de idade menor (50,2 anos), traumas de alta energia (54%), e fraturas tipo C (73%), enquanto no grupo 2 fraturas tipo A prevaleceram (72%). As radiografias apresentaram diferença significativa quanto à média de valores de inclinação radial (21,5° no grupo 1 e 16,5° no grupo 2 [ *p* < 0,001] nas mulheres e, 21,3° no grupo 1 e 17° no grupo 2 [ *p* < 0,001] nos homens) e inclinação volar (10,1° e 12,8° no grupo 1 e 2, respectivamente [ *p* < 0,001]), bem como no número absoluto de casos que reestabeleceram os parâmetros anatômicos nas três variáveis avaliadas, sendo todos melhores no grupo 1.

**Conclusão:** Os cirurgiões de mão trataram as fraturas mais graves e apresentaram os melhores resultados radiográficos.

## Introdução

A fratura de rádio distal (FRD) é a mais comum do membro superior, 1 2 3 4 5 6 7 8 9 10 representando entre 10 e 25% das lesões na emergência ortopédica. 11 12 13 14 15 Possui distribuição bimodal, associada a traumas de alta energia em jovens e de baixa energia em idosos. 2 4 7 A incidência vem crescendo devido ao aumento da expectativa de vida e da osteoporose. 4 6 7 9

Em idosos é a segunda fratura mais comum, 16 comprometendo funcionalmente e incapacitando esses pacientes. 13 Verifica-se, também, o impacto nos pacientes mais jovens, 12 influenciando-os financeiramente e profissionalmente, tornando-se um problema de saúde pública. 16

O objetivo do ortopedista é restaurar a função e mobilidade do punho. 2 3 16 Porém, não existe consenso sobre o tratamento ideal, devendo ser levados em consideração: idade, grau de atividade, ocupação, qualidade óssea, tipo de fratura e qualidade de vida. 1 2 10 12 16

É difícil avaliar o resultado do tratamento, uma vez que o sucesso terapêutico é multifatorial. O ortopedista pode interferir no restabelecimento dos parâmetros radiográficos. 5 7 Portanto, restaurá-los é imprescindível para alcançar resultados funcionais satisfatórios. 4 11 13 17

Os parâmetros radiográficos mais importantes são: altura radial (AR), inclinação radial (IR), e inclinação volar (IV). 4 5 11 Entretanto, não existe consenso sobre os valores ideais. 10 Assim, consideramos os seguintes valores: AR (11,6 mm ± 1,6 mm), IR (mulheres: 24,7° ± 2,5°, e homens: 22,5° ± 2,1°) e IV (11,2° ± 4,6°). 18

O tratamento conservador apresenta resultados ruins, sobretudo em jovens com fratura articular. 1 2 Nos idosos, este método não é capaz de manter a redução; todavia, não se observa diferença na capacidade funcional entre o tratamento conservador ou cirúrgico. 4 11 12

As alternativas para tratamento cirúrgico são: fio de Kirschner (FK), fixador externo (FE) e redução aberta com fixação interna (RAFI). 2 4 As técnicas do FK e FE são menos invasivas, menos complexas e causam menor impacto financeiro. Porém, a RAFI constitui-se como o tratamento mais efetivo para fraturas instáveis e articulares, 2 pois promove uma fixação estável e mobilização precoce 4 15 ; melhores resultados funcionais e satisfação 6 8 9 16 17 e, menores índices de osteoartrose. 15

Neste sentido, a perda da AR é um fator relevante que afeta a capacidade funcional, podendo acarretar dor, osteoartrose, instabilidade e prono-supinação limitada. 17 Além disso, a IV com desvio dorsal acima de 20° aumenta o risco de osteoartrose. 4 16 Por fim, o nível de satisfação do paciente relaciona-se com a quantidade de dor, e a função e mobilidade do punho após o tratamento. 19 20

Questiona-se se os ortopedistas têm recebido treinamento suficiente para tratar este tipo de fratura. Um estudo recente, com diretores de programas de cirurgia da mão, demonstrou que a prática das técnicas de RAFI é essencial na formação; entretanto, existe uma lacuna de exposição de até 53%. 21

Tendo em vista o déficit na formação e a relevância do tema, este trabalho tem como objetivo avaliar as variáveis sociodemográficas e radiográficas das FRD, bem como comparar os resultados entre os cirurgiões especialistas em cirurgia da mão e ortopedistas não especialistas.

## Materiais e métodos

Trata-se de uma coorte retrospectiva realizada com pacientes com FRD atendidos em um hospital de trauma no sul do Brasil.

A amostra possui 200 pacientes atendidos entre 01/01/2020 e 31/12/2020. Desse total, a metade dos casos foram tratados por especialistas em cirurgia da mão (grupo 1) e a outra metade por ortopedistas não especialistas (grupo 2).

Foram incluídos os 100 últimos pacientes atendidos por cada grupo no período mencionado. Também foram incluídos: pacientes acima de 18 anos, com fratura unilateral tratados em até uma semana do trauma.

O único critério de exclusão foi pacientes que não preencheram os critérios de inclusão. Nenhum paciente foi excluído por falta de dados em prontuário.

As variáveis analisadas em prontuário foram: idade, sexo, mecanismo de trauma, lateralidade, presença de comorbidades e fraturas associadas, e método de tratamento.

As radiografias do dia do trauma foram utilizadas para classificar as fraturas pela classificação Arbeitsgemeinschaft für Osteosynthesefragen (AO). 22

As radiografias de controle foram realizadas após uma semana da imobilização gessada ou do procedimento cirúrgico. As radiografias foram utilizadas para calcular a AR, a IR e a IV.

Os critérios utilizados para indicação do tratamento cirúrgico foram a presença de sinais de instabilidade da fratura (desvio dorsal > 20°, cominuição da cortical dorsal, acometimento articular, fratura da ulna associada, encurtamento do rádio > 9 mm). 1

Os parâmetros radiográficos foram coletados pelo mesmo pesquisador. Foi considerado para a análise da IR, na radiografia em anteroposterior, o ângulo entre uma linha perpendicular à linha do eixo longo da diáfise do rádio e uma linha que liga o estiloide radial ao canto ulnar do rádio. Já a AR, também avaliada na radiografia em anteroposterior, apresenta-se na diferença do comprimento axial entre a ponta do estiloide radial e o canto ulnar do rádio. A inclinação volar, avaliada na radiografia em perfil, foi definida pelo ângulo formado entre uma perpendicular ao eixo longitudinal do rádio e uma linha formada pela conexão do ápice da borda volar e dorsal do rádio. 18

Os parâmetros considerados normais foram: 11,6mm ± 1,6mm para AR; 24,7° ± 2,5° para IR nas mulheres e 22,5° ± 2,1° nos homens e; 11,2° ± 4,6° de IV. 18

Os dados foram processados em um microcomputador, no banco de dados Microsoft Excel 2016 (Microsoft Corp., Redmond, WA, EUA). Para a descrição de variáveis quantitativas foram utilizados a média, os valores mínimo e máximo e o desvio padrão. Para a sumarização de variáveis qualitativas foram considerados frequências e percentuais. Para a comparação dos grupos em relação a variáveis quantitativas foi considerado o teste t de Student. A avaliação da associação entre duas variáveis qualitativas foi realizada considerando-se o teste do qui-quadrado ou o teste exato de Fisher. A análise estatística foi obtida com o auxílio do programa SPSS Statistics for Windows, versão 18.0 (SPSS Inc., Chicago, IL, EUA) e o nível de significância utilizado foi de 5%.

Por fim, este estudo foi aprovado pelo Comitê de Ética em Pesquisa sob o parecer n° 5.310.541.

## Resultados

A amostra global ( Tabela 1 ) é composta, em sua maioria, por mulheres (54%), com idade média de 50,7 anos. A FRD teve maior prevalência no lado esquerdo (53%), o mecanismo de baixa energia foi o mais comum. Destas, 86 pacientes tiveram fraturas tipo A (extra-articular), 25 do tipo B (articular parcial) e 89 do tipo C (articular completa). O método de tratamento mais utilizado foi o FK (62,5%), seguido de RAFI, conservador e FE.

**Tabela 1:** Dados dos pacientes tratados pelos especialistas e não especialistas em cirurgia da mão

		Especialista (grupo 1)	Não Especialista (grupo 2)	*p*
Sexo	**Masculino (n)**	48	44	0,57
	**Feminino (n)**	52	56	
Idade	**Média (anos)**	50,2	51,3	0,64
Mecanismo	**Baixa energia (n)**	46	70	0,001
	**Alta energia (n)**	54	30	
Lateralidade	**Direita (n)**	49	45	0,571
	**Esquerda (n)**	51	55	
Comorbidade	**Sim (n)**	28	33	0,443
	**Não (n)**	72	67	
Fratura associada	**Sim (n)**	24	12	0,027
	**Não (n)**	76	88	
Classificação AO	**A (n)**	14	72	< 0,001
	**B (n)**	13	12	
	**C (n)**	73	16	
Altura radial	**Média (mm)**	11,04	10,8	0,299
	**Perda (n)**	11	35	< 0,001
	**Ganho (n)**	3	12	
Inclinação radial	**Mulheres (°)**	21,5	16,5	< 0,001
	**Homens (°)**	21,3	17	< 0,001
	**Perda (n)**	51	87	< 0,001
	**Ganho (n)**	3	2	
Inclinação volar	**Média (°)**	10,1	12,8	< 0,001
	**Perda (n)**	16	1	< 0,001
	**Ganho (n)**	5	15	
Tratamento	**Fio de Kirschner (n)**	47	78	–
	**Placa e parafuso (n)**	39	15	
	**Gesso (n)**	9	5	
	**Fixador externo (n)**	5	2	

Legenda: n – número de casos, mm – milímetros, ° - graus, p – comparação entre os grupos.

Os parâmetros radiográficos apresentaram média de 10,92 mm na AR. Mulheres apresentaram IR média de 18,9° e homens de 19,5°, e, por fim, uma IV de 11,4°. Destes pacientes, 23% perderam e 7,5% ganharam AR; 69% perderam e 2,5% ganharam IR e; 8,5% perderam e 10% ganharam IV.

Avaliando apenas os pacientes tratados pelos especialistas em cirurgia da mão (grupo 1 - Tabela 1 ), foi encontrada prevalência do mecanismo de alta energia (54%) e fraturas tipo C (73%). Os parâmetros radiográficos foram: AR de 11,04 mm (8–15,36, DP: 1,17), IR de 21,5° (16–27, DP: 2,4) nas mulheres e de 21,3° (17–28, DP: 2,7) nos homens, e IV de 10,1° (2–22, DP: 3,8). Destes, apenas 11 casos apresentaram perda e 3 apresentaram ganho de AR; 51 casos apresentaram perda e 3 apresentaram ganho na IR; e, 16 perderam e 5 ganharam IV.

Com relação aos casos tratados pelos ortopedistas não especialistas em cirurgia da mão (grupo 2 - Tabela 1 ), obtivemos o mecanismo de baixa energia em 70% dos casos, e a fratura mais comum foi do tipo A (72%). Neste grupo encontrou-se média da AR de 10,8 mm (5,95–15,71, DP:1), IR de 16,5° (10–29, DP: 3,8) nas mulheres e de 17° (12–25, DP: 2,97) nos homens, e IV de 12,8° (3–27, DP: 3,5). Destes pacientes, 35 perderam e 12 ganharam AR; 87 perderam e 2 ganharam IR e; 1 perdeu e 15 ganharam IV.

Ao comparar os grupos ( Tabela 1 ), encontrou-se mais trauma de alta energia no grupo 1 (54 × 30 casos, *p* < 0,001), bem como de fraturas associadas (24 × 12 casos, *p* = 0,027). O grupo 2 teve predomínio nas fraturas do tipo A (72%), enquanto as fraturas do tipo C foram predominantes no grupo 1 (73%).

Comparando os parâmetros radiográficos, encontramos que a média alcançada da AR não apresentou diferença estatística (11,04 vs 10,8, *p* = 0,29), enquanto as médias de IR e IV foram melhores no grupo 1, sendo a IR média nas mulheres de 21,5° vs 16,5° ( *p* < 0,001) e de 21,3° vs 17° ( *p* < 0,001) nos homens, e IV de 10,1° e 12,8° nos grupos 1 e 2, respectivamente ( *p* < 0,001). Quando avaliamos o número absoluto de casos que não alcançaram os parâmetros radiográficos, houve diferença estatística entre eles, sendo piores os resultados do grupo 2 ( *p* < 001).

Conforme o tipo de fratura, as do tipo A ( Tabela 2 ) tiveram melhores resultados de IR e IV, com média de IR de 22,2° vs 16,5° ( *p* < 0,001) nas mulheres, e de 22° vs 17,7° ( *p* = 0,011) nos homens, e média de IV de 10,4° vs 12,5° ( *p* = 0,017).

**Tabela 2:** Comparação das fraturas tipo A entre os grupos tratados por especialistas em cirurgia da mão e não especialistas

		Tipo A		
		Especialista (grupo 1)	Não Especialista (grupo 2)	*p*
Sexo	**Masculino (n)**	4	31	0,313
	**Feminino (n)**	10	41	
Idade	**Média (anos)**	49,1	51,8	0,61
Mecanismo	**Baixa energia (n)**	8	52	0,261
	**Alta energia (n)**	6	20	
Lateralidade	**Direita (n)**	8	31	0,333
	**Esquerda (n)**	6	41	
Comorbidade	**Sim (n)**	3	22	0,491
	**Não (n)**	11	50	
Fratura associada	**Sim (n)**	2	8	0,735
	**Não (n)**	12	64	
Altura radial	**Média (mm)**	10,91	10,73	0,654
	**Perda (n)**	1	26	–
	**Ganho (n)**	0	9	
Inclinação radial	**Mulheres (°)**	22,2	16,5	< 0,001
	**Homens (°)**	22	17,7	0,011
	**Perda (n)**	8	63	–
	**Ganho (n)**	0	2	
Inclinação volar	**Média (°)**	10,4	12,5	0,017
	**Perda (n)**	1	0	–
	**Ganho (n)**	0	10	
Tratamento	**Fio de Kirschner (n)**	12	65	–
	**Placa e parafuso (n)**	1	2	
	**Gesso (n)**	0	5	
	**Fixador externo (n)**	1	0	

Legenda: n – número de casos, mm – milímetros, ° - graus, p – comparação entre os grupos.

As fraturas do tipo B ( Tabela 3 ) apresentaram melhores resultados apenas na IR em mulheres do grupo 1 (21° vs 15,8°, *p* = 0,015). Por fim, as fraturas do tipo C ( Tabela 4 ) apresentaram melhores resultados na IR (nas mulheres 21,3° vs 17,3°, *p* = 0,001 e nos homens 21,4° vs 16,7°, *p* < 0,001) e na IV (10° vs 13,1°, *p* = 0,02) no grupo 1, sendo que nessas fraturas o tratamento de preferência foi a RAFI em ambos os grupos.

**Tabela 3:** Comparação das fraturas tipo B entre os grupos tratados por especialistas em cirurgia da mão e não especialistas

		Tipo B		
		Especialista (grupo 1)	Não Especialista (grupo 2)	*p*
Sexo	**Masculino (n)**	9	4	0,073
	**Feminino (n)**	4	8	
Idade	**Média (anos)**	41,8	48,3	0,422
Mecanismo	**Baixa energia (n)**	3	8	0,028
	**Alta energia (n)**	10	4	
Lateralidade	**Direita (n)**	7	6	0,848
	**Esquerda (n)**	6	6	
Comorbidade	**Sim (n)**	3	5	0,319
	**Não (n)**	10	7	
Fratura associada	**Sim (n)**	5	1	0,078
	**Não (n)**	8	11	
Altura radial	**Média (mm)**	11,32	11,19	0,85
	**Perda (n)**	0	3	–
	**Ganho (n)**	1	2	
Inclinação radial	**Mulheres (°)**	21	15,8	0,015
	**Homens (°)**	20,4	18,3	0,235
	**Perda (n)**	6	10	–
	**Ganho (n)**	1	0	
Inclinação volar	**Média (°)**	10,7	12,3	0,145
	**Perda (n)**	1	0	–
	**Ganho (n)**	1	1	
Tratamento	**Fio de Kirschner (n)**	3	8	–
	**Placa e parafuso (n)**	4	3	
	**Gesso (n)**	6	0	
	**Fixador externo (n)**	0	1	

Legenda: n – número de casos, mm – milímetros, ° - graus, p – comparação entre os grupos.

**Tabela 4:** Comparação das fraturas tipo C entre os grupos tratados por especialistas em cirurgia da mão e não especialistas

		Tipo C		
		Especialista (grupo 1)	Não especialista (grupo 2)	*p*
Sexo	**Masculino (n)**	35	9	0,547
	**Feminino (n)**	38	7	
Idade	**Média (anos)**	51,9	51,6	0,947
Mecanismo	**Baixa energia (n)**	35	10	0,292
	**Alta energia (n)**	38	6	
Lateralidade	**Direita (n)**	34	8	0,804
	**Esquerda (n)**	39	8	
Comorbidade	**Sim (n)**	22	6	0,566
	**Não (n)**	51	10	
Fratura associada	**Sim (n)**	17	3	0,694
	**Não (n)**	56	13	
Altura radial	**Média (mm)**	11,02	10,8	0,649
	**Perda (n)**	10	6	–
	**Ganho (n)**	2	1	
Inclinação radial	**Mulheres (°)**	21,3	17,3	0,001
	**Homens (°)**	21,4	16,7	< 0,001
	**Perda (n)**	37	14	–
	**Ganho (n)**	2	0	
Inclinação volar	**Média (°)**	10	13,8	0,02
	**Perda (n)**	14	1	–
	**Ganho (n)**	4	4	
Tratamento	**Fio de Kirschner (n)**	32	5	–
	**Placa e parafuso (n)**	34	10	
	**Gesso (n)**	3	0	
	**Fixador externo (n)**	4	1	

Legenda: n – número de casos, mm – milímetros, ° - graus, p – comparação entre os grupos.

## Discussão

A literatura demonstra que FRD acomete principalmente mulheres, 3 4 5 6 7 8 9 10 11 12 13 14 15 17 como verificamos neste trabalho. A média de idade foi de 50,7 anos, inferior à da maioria dos trabalhos, 2 3 4 5 6 7 9 12 13 15 18 sendo que poucos demonstraram médias inferiores à encontrada. 8 10

A FRD possui distribuição bimodal, com os traumas de alta energia associados a pacientes mais jovens (< 60 anos). 4 8 Entretanto, no presente trabalho, a média de idade esteve abaixo dos 60 anos, porém mecanismos traumas foram de baixa energia em sua maioria. Uma possibilidade para explicar essa divergência diz respeito à grande variação de idade dos participantes desta amostra, variando de 19 a 100 anos.

A lateralidade é importante visto o impacto nas atividades cotidianas do indivíduo. 12 13 Alguns autores demonstram predominância no membro não dominante 3 8 13 e outros no dominante. 5 6 11 14 Outros utilizam-se da lateralidade como direita e esquerda. Nestes trabalhos, as fraturas ocorreram predominantemente no membro esquerdo, 2 3 9 15 assim como em nosso estudo.

Na presente pesquisa, apenas 30,5% dos pacientes apresentavam comorbidades, corroborando demais estudos que trouxeram baixa ocorrência de comorbidades. 5 10 Encontrou-se 18% de fraturas associadas e a literatura indica que de 39 a 84% dos casos tem algum tipo de lesão associada, não especificando o percentual de fraturas associadas. 12

Em relação ao tipo de fratura, 86 dos casos eram de fraturas do tipo A, 25 do tipo B e 89 do tipo C; o que condiz com outros trabalhos, os quais evidenciaram maior ocorrência de fraturas dos tipos A 11 12 e C. 4 6 9 15

A literatura mostra que os tratamentos mais utilizados são a RAFI e o método conservador. Os que predominam tratamento conservador 10 11 12 advogam que este é o principal método, tendo em vista a grande remodelação óssea em jovens e a baixa demanda funcional nos idosos. 10 Já os artigos nos quais há preferência pela RAFI 2 3 6 17 referem que este método proporcionaria melhor redução da fratura, possibilitando mobilidade precoce e melhores resultados funcionais, 6 8 9 14 15 16 17 além de demonstrarem que cirurgiões de mão são mais propensos a utilizar este método. 16 Apesar disso, em nosso estudo, o FK foi o método de preferência (62,5%); possivelmente pelo alto número de fraturas extra-articulares, associado ao alto número de idosos, para quem um método menos invasivo seria o ideal. Além disso, a maioria dos estudos foram realizados em países desenvolvidos, nos quais os métodos menos agressivos vêm sendo substituídos por fixação com placa bloqueada 10 ; portanto, nós podemos, ainda, estar neste período de transição.

Considerando o grupo 1, observou-se que a maioria dos casos foi ocasionada por mecanismo de alta energia e que a média de idade foi ligeiramente menor neste grupo. Isto corrobora a ideia de que os mais jovens se associam com mecanismos de alta energia. Além disso, neste grupo, houve predomínio de fraturas do tipo C (73%), o oposto do outo grupo, nos qual as fraturas tipo A predominaram (72%), provavelmente pelos casos mais complexos serem manejados por especialistas em cirurgia da mão. 4

Com relação aos parâmetros radiográficos, os artigos diferem na forma de apresentação dos resultados, sendo apresentados os valores absolutos após o tratamento, 2 3 5 8 14 17 ou a variação pré- e pós-tratamento. 4 9 11 12 Em nosso estudo, foi encontrada uma média de AR de 10,92 mm, que converge com a literatura. 3 5 8 14 17 A IR atingida em nossa amostra foi de 18,9° nas mulheres e 19,5° nos homens e a IV de 11,4°; estes dados ficaram abaixo do encontrado na literatura. 2 3 4 5 7 8 9 11 14 O método de fixação pode ser um dos responsáveis pela diferença constatada, embora os estudos tenham demonstrado não existir diferença dos resultados radiográficos relacionados com o método de fixação. 1 2

Ademais, há contradição na relação entre os resultados radiográficos e a função, principalmente em idosos cuja demanda é menor. 3 4 5 6 7 11 13 Entretanto, diversos fatores influenciam o resultado do tratamento, dentre eles a redução da fratura que o ortopedista pode interferir. 5 7 Portanto, a redução é essencial para que se atinja melhores resultados funcionais, 4 11 13 17 com menor taxa de osteoartrose 6 15 e melhor mobilidade. 4

Um estudo demonstrou que a osteoartrose pós-traumática se relaciona com alterações radiográficas como IR e IV. 15 Outro trabalho demonstrou que apenas 54% dos casos restabelecem todos os parâmetros radiográficos, sendo a perda da AR responsável pelos piores resultados funcionais, perda da mobilidade, diminuição da força de preensão e dor crônica. 4 6 17

Comparando os parâmetros radiográficos entre os grupos, encontramos que no grupo 1, o número de casos que restabeleceram os parâmetros de normalidade foi maior que no grupo 2 e as médias de IR e IV foram melhores no grupo 1. Não foi encontrada, na base de dados pesquisada, comparação semelhante à do presente estudo. Um trabalho referiu não existir diferença nos resultados quando considerada a experiência do cirurgião. 4 Tendo em vista que uma melhor redução pode levar ao melhor resultado funcional e de satisfação, podemos sugerir que os pacientes tratados por especialistas em cirurgia da mão tiveram resultados mais vantajosos em nosso estudo.

Considerando o tipo de fratura, as do tipo A tiveram um maior percentual de pacientes que recuperaram a AR no grupo 1; as médias da IR e IV também foram melhores neste grupo. As fraturas tipo B tiveram melhor resultado no grupo 1 no quesito média de IR nas mulheres. Enquanto as fraturas do tipo C apresentaram melhores resultados na IR e IV quando tratados por especialistas em cirurgia da mão.

## Limitações do Estudo

Além deste estudo ser retrospectivo, outras limitações foram: a falta de consenso sobre os parâmetros radiológicos considerados normais 9 ; as radiografias utilizadas foram de uma semana pós-tratamento instituído, não considerando possíveis complicações ou perda de redução a longo prazo. Os casos tratados pelos especialistas em cirurgia da mão, na maioria das vezes, são mais complexos, sendo difícil colher amostras homogêneas entre os grupos. Apesar dos pacientes de baixa demanda aceitarem um maior desvio da fratura de rádio distal para o tratamento não cirúrgico, o grau funcional dos pacientes pré- e pós-tratamento não foi avaliado. Portanto, são necessários novos estudos funcionais e de satisfação para determinar o resultado do tratamento, visto que a redução da fratura é apenas um dos pilares para o sucesso terapêutico.

## Conclusão

Este estudo demonstrou que os especialistas em cirurgia da mão tratam os casos mais complexos de FRD. E, mesmo se tratando de casos mais graves, os especialistas apresentaram melhores resultados radiográficos quando comparados aos ortopedistas não especialistas.

Visto que estes resultados estão diretamente ligados com melhores resultados funcionais e de satisfação do paciente, os ortopedistas devem estar preparados para alcançarem os melhores resultados radiográficos.
